# Multi-omics and experimental validation reveal ISG20L2 as a potential pro-oncogenic regulator in gastric cancer

**DOI:** 10.3389/fonc.2026.1906513

**Published:** 2026-09-16

**Authors:** Song Liang, Chuanjun Wang, Yongkang Liang, Chuansi Wang, Xiuming Lu, Shenwei Wu, Changyu He, Weiwei Yuan

**Affiliations:** 1 Department of General Surgery, Lu’an Affiliated Hospital of Anhui Medical University, Lu’an, Anhui, China; 2 Department of General Surgery, Ruijin Hospital, Shanghai Jiao Tong University, School of Medicine, Shanghai, China; 3 Department of Thyroid Surgery, Baoshan Hospital Affiliated to Shanghai University of Traditional Chinese Medicine, Shanghai, China

**Keywords:** biomarker, CD8+ T-cell exhaustion, cell cycle, gastric cancer, immune microenvironment, ISG20L2

## Abstract

**Background:**

Gastric cancer is a major malignancy with molecular heterogeneity and limited biomarkers for risk stratification and therapeutic guidance. Interferon-stimulated 20 kDa exonuclease-like 2 (ISG20L2), a regulator of ribosome biogenesis, has been implicated in tumor progression, but its relevance in gastric cancer is poorly defined. This study aimed to characterize the potential pro-oncogenic and immune relevance of ISG20L2 in gastric cancer.

**Methods:**

Public multi-omics datasets and two single-cell RNA-sequencing cohorts were used to evaluate ISG20L2 expression and its associations with molecular features, tumor functional states, immune infiltration, and immunotherapy-related characteristics. Protein expression was validated in gastric cancer cell lines and nine paired clinical tissues, and functional effects of ISG20L2 knockdown were assessed in AGS and HGC-27 cells.

**Results:**

ISG20L2 was significantly upregulated in gastric cancer and inversely correlated with promoter methylation. Single-cell analysis showed predominant enrichment of ISG20L2 in exhausted CD8+ T cells. High ISG20L2 expression was closely associated with cell-cycle activity, immune infiltration, PD-L1 expression and predicted immunotherapy response. Experimentally, ISG20L2 protein was elevated in gastric cancer cell lines and tumor tissues. ISG20L2 knockdown markedly suppressed proliferation, clonogenicity, migration and invasion in both AGS and HGC-27 cells.

**Conclusion:**

ISG20L2 is aberrantly upregulated and associated with malignant biological features and immune-related characteristics in gastric cancer, supporting its potential role as a pro-oncogenic regulator and a candidate molecular indicator requiring further clinical validation.

## 1 Introduction

Gastric cancer remains a major global health burden and a leading cause of cancer-related mortality, particularly in East Asia (1, 2). Despite advances in multimodal therapies, the prognosis of gastric cancer remains unfavorable, largely owing to substantial tumor heterogeneity, late diagnosis, and the absence of reliable molecular biomarkers for risk stratification (3–5). Currently available biomarkers, including HER2, PD-L1, and microsatellite instability/deficient mismatch repair (MSI/dMMR), are applicable to only a subset of patients and inadequately reflect the molecular complexity of gastric cancer, highlighting the need to identify additional molecular regulators (6, 7). Beyond established clinical markers, diverse molecular biomarkers have been explored in gastric cancer. Non-coding RNAs, including microRNAs (miRNAs), long non-coding RNAs (lncRNAs), and circular RNAs (circRNAs), have potential roles in early detection, stratification, prognosis, and tumor-microenvironment assessment (8–10). Protein biomarkers, including KCND2 and CNTN1, are associated with tumor grade, invasion, immune features, and clinical outcomes (11, 12). Biosensing and blood-based approaches have also enabled sensitive detection of miR-196b/miR-221, Vimentin, and circulating miRNA/lymphocyte signatures (13–15). However, these candidates reflect specific aspects of tumor biology and require prospective validation before clinical use. Interferon-stimulated 20 kDa exonuclease-like 2 (ISG20L2) is a nucleolus-localized 3′–5′ exoribonuclease involved in precursor ribosomal RNA (pre-rRNA) processing and ribosome biogenesis, thereby maintaining protein synthesis homeostasis (16, 17). Dysregulated ribosome biogenesis is increasingly recognized as a feature of tumor growth and progression, suggesting that molecules involved in this process may have biological and translational relevance in cancer. Emerging evidence indicates that ISG20L2 is aberrantly upregulated in multiple solid tumors, including hepatocellular carcinoma, breast cancer, and lung adenocarcinoma, and is closely associated with tumor progression and poor prognosis (18–20). Functional and mechanistic studies further suggest that ISG20L2 promotes malignant phenotypes, such as cancer cell proliferation, migration, and invasion, potentially through the regulation of transcriptional programs or ribosome biogenesis–related pathways (16–18, 21).

However, the expression pattern, biological function, and clinical relevance of ISG20L2 in gastric cancer remain largely unexplored. In the present study, we integrated public multi-omics datasets, two independent single-cell RNA-sequencing cohorts, clinical tissue validation, and *in vitro* functional assays to evaluate ISG20L2 expression and its associations with molecular features, cell-cycle activity, and the tumor immune microenvironment. We further investigated the functional impact of ISG20L2 on gastric cancer progression. Our findings suggest that ISG20L2 may represent a potential pro-oncogenic regulator associated with gastric cancer biology, while its clinical utility requires further validation in well-characterized cohorts.

## 2 Materials and methods

### 2.1 Public datasets and expression analysis

Transcriptomic data of ISG20L2 across human cancers were obtained from The Cancer Genome Atlas (TCGA, https://portal.gdc.cancer.gov/) and the Genotype-Tissue Expression (GTEx, https://gtexportal.org/) databases. Integrated TCGA-GTEx expression matrices were downloaded from the UCSC Xena Toil RNA-seq Recompute Compendium, including “tcga_RSEM_gene_tpm” and “gtex_RSEM_gene_tpm”. Since these matrices are provided as log2-transformed TPM values, no further logarithmic transformation was performed. TCGA primary tumor, TCGA adjacent normal, and GTEx normal samples were merged at the gene level for each cancer type.

Batch effects between TCGA and GTEx were corrected separately for each cancer type using the empirical Bayes ComBat algorithm implemented in the “sva” package (R version 4.3.2). Data source (TCGA or GTEx) was defined as the batch variable, while tumor/normal status was included as a covariate to preserve biological differences. ComBat correction was performed only for cancer types with sufficient TCGA and GTEx normal samples. Corrected ISG20L2 expression values were standardized as Z scores [Z = (x − μ)/σ], and samples with |Z| > 3 were excluded as outliers according to the downstream analysis criteria. Differential expression between tumor and normal tissues was evaluated using the Wilcoxon rank-sum test. Batch correction was assessed by principal component analysis (PCA) based on the 1,000 most variable genes before and after ComBat correction, with samples annotated by data source and tumor/normal status. TCGA-only pan-cancer analyses were conducted independently without cross-study batch correction. Paired ISG20L2 expression analysis in STAD was performed using matched tumor and adjacent normal tissues from the same patients. Associations between ISG20L2 expression and MSI status were evaluated using TCGA-STAD molecular subtype data from UCSC Xena and immune subtype annotation files. Promoter methylation analysis was performed using TCGA methylation datasets and Spearman correlation analysis. All statistical analyses and visualizations were performed in R version 4.3.2. The selected bioinformatics analysis scripts used in this study are provided in the **Supplementary Materials**.

### 2.2 Single-cell RNA-sequencing analysis

To determine the cellular distribution of ISG20L2 in gastric cancer, two independent single-cell RNA-sequencing datasets, STAD_GSE134520 and STAD_GSE167297, were downloaded from the Gene Expression Omnibus (GEO, https://www.ncbi.nlm.nih.gov/geo/). Raw single-cell data were processed using the Seurat package (version 5.0.1) in R version 4.3.2, including quality control, normalization, scaling, and batch-effect correction. Dimensionality reduction was performed using uniform manifold approximation and projection (UMAP). Cell populations were annotated according to established marker genes and available annotation information provided in the original datasets, including T cells, B cells, malignant cells, NK cells, and macrophages. ISG20L2 expression was quantified across annotated cell populations, and average expression values were calculated for each cell type. FeaturePlot and DotPlot functions in Seurat, together with ggplot2, were used for visualization. Particular attention was given to the exhausted CD8+ T-cell subset (CD8Tex), in which ISG20L2 expression was highest.

### 2.3 Functional state analysis

The association between ISG20L2 expression and tumor functional states was evaluated using the CancerSEA database (http://biocc.hrbmu.edu.cn/CancerSEA/). Pearson correlation analysis was performed to assess the relationship between ISG20L2 expression and multiple cancer-related functional states, including angiogenesis, apoptosis, differentiation, epithelial–mesenchymal transition (EMT), hypoxia, inflammation, invasion, proliferation, and stemness. Correlation analyses and visualizations were conducted using R version 4.3.2 with the “ggplot2”, “ggpubr”, “tidyr”, and “randomcoloR” packages. In addition, the association between ISG20L2 expression and cell-cycle functional state scores in STAD was evaluated using Pearson correlation analysis.

### 2.4 Signaling pathway and enrichment analyses

To investigate biological pathways associated with ISG20L2, pathway enrichment analyses were conducted across common tumor types using TCGA transcriptomic datasets. Gene set enrichment analysis (GSEA) and gene set variation analysis (GSVA) were performed in gastric cancer using the “clusterProfiler”, “GSVA”, and “fgsea” packages in R version 4.3.2. Hallmark and KEGG gene sets were obtained from the Molecular Signatures Database (MSigDB, https://www.gsea-msigdb.org/). Samples were stratified into high- and low-ISG20L2 expression groups according to median expression levels, and differentially expressed genes between the two groups were subjected to enrichment analyses. Pathways enriched in the high- and low-expression groups were identified. Correlation analyses between ISG20L2 expression and cell-cycle activity, as well as differential expression across cell-cycle phases, were performed using Pearson correlation analysis and one-way ANOVA.

### 2.5 Immune infiltration and immunoregulatory analyses

To explore the immune relevance of ISG20L2 in gastric cancer, multiple immune deconvolution algorithms, including TIMER, CIBERSORT, EPIC, xCell, and MCP-counter, were used to estimate infiltrating immune-cell populations. Immune infiltration analyses were conducted using the TIMER database (https://cistrome.shinyapps.io/timer/), ESTIMATE platform (https://bioinformatics.mdanderson.org/estimate/), and corresponding R scripts. Spearman correlation analysis was performed to assess the association between ISG20L2 expression and immune infiltration levels. Heatmaps were generated using the “pheatmap” and “ggplot2” packages to visualize the correlations between ISG20L2 and immune-related features across cancers, including proliferative versus activated T-cell ratios and PD-L1 expression in STAD. Methylation of tumor-infiltrating lymphocytes (MeTIL) analysis was used to compare the infiltration levels of T cells, NK cells, B cells, and regulatory T cells (Tregs) between high- and low-ISG20L2 expression groups. TIP analysis (Tumor Immunophenotype Profiling, http://biocc.hrbmu.edu.cn/TIP/) was further performed to evaluate the association between ISG20L2 expression and anti-tumor immune process scores.

### 2.6 Genomic alteration, virtual knockout and immunotherapy-response analyses

To assess the genomic and therapeutic relevance of ISG20L2, mutation landscape analyses were performed using TCGA mutation datasets and the cBioPortal platform (https://www.cbioportal.org/). Mutation annotation format (MAF) files were analyzed using the “maftools” R package to characterize genomic alteration patterns associated with ISG20L2 in gastric cancer. Virtual knockout analyses based on TCGA transcriptomic data were conducted to simulate ISG20L2 depletion and identify downstream transcriptional changes. Correlation analyses between ISG20L2 and selected genes of interest were performed using Pearson correlation analysis. To evaluate potential clinical significance, immunotherapy-related analyses were performed using the Tumor Immune Dysfunction and Exclusion (TIDE) platform (http://tide.dfci.harvard.edu/), The Cancer Immunome Atlas (TCIA) database (https://tcia.at/), and ImmuCellAI platform (https://guolab.wchscu.cn/ImmuCellAI/). Statistical analyses and visualization were performed in R version 4.3.2 using the “ggplot2”, “survival”, and “survminer” packages.

### 2.7 Clinical tissue samples and cell culture

Nine paired STAD tissues and matched adjacent non-tumor tissues were collected from patients who underwent surgical resection at the Affiliated Lu’an Hospital of Anhui Medical University between April 2025 and August 2025. None of the patients received preoperative radiotherapy, chemotherapy or immunotherapy. All specimens were pathologically confirmed, immediately frozen in liquid nitrogen and stored at −80 °C until protein extraction. The study was approved by the Ethics Committee of the Affiliated Lu’an Hospital of Anhui Medical University [Ethics approval No.:2026LLKS-KY-045], and written informed consent was obtained from all participants. Human gastric cancer cell lines and the normal gastric epithelial cell line GES-1 were obtained from the American Type Culture Collection (ATCC, USA). Cells were cultured in DMEM (Gibco, USA) supplemented with 10% fetal bovine serum (Gibco, USA), 100 units/mL penicillin and 100 μg/mL streptomycin, and maintained at 37 °C in a humidified incubator with 5% CO_2_.

### 2.8 Western blot analysis

Western blotting was performed to evaluate endogenous ISG20L2 protein expression in gastric cancer cell lines and clinical tissues and to verify ISG20L2 knockdown efficiency in AGS and HGC-27 cells. Total protein was extracted from cultured cells or tissue samples using RIPA lysis buffer containing PMSF and phosphatase inhibitors (Meilunbio, China; MA0151 and MB12707), and protein concentrations were determined using a BCA protein assay kit (GBCBIO, China; G3422/G3522). Equal amounts of 40 μg protein per lane were separated by SDS-PAGE and transferred onto 0.45 μm PVDF membranes (Millipore, USA; IPVH00010) using an eBlot™ L1 rapid wet transfer system (GenScript, China; L00686C), as described previously with minor modifications. After blocking with 5% non-fat milk in TBST for 2 h at room temperature, the membranes were incubated overnight at 4 °C with primary antibodies against ISG20L2 (1:1000; Proteintech, China) and Tubulin (1:1000; fsbio-mall, China). After washing, the membranes were incubated with HRP-conjugated goat anti-rabbit secondary antibody (1:10000; Proteintech, China; RGAR001) for 2 h at room temperature. Protein bands were visualized using an enhanced chemiluminescence substrate (ECL; Affinity, China; KF8003) and detected with a chemiluminescence imaging system (Hangzhou Shenhua Technology, China; SH-523). Band intensities were quantified using ImageJ software, with Tubulin used as the internal loading control.

### 2.9 Construction of ISG20L2 knockdown cell models

ISG20L2 knockdown models were established in AGS and HGC-27 gastric cancer cells using pLVX-shRNA2-Puro-based shRNA plasmids. Three shRNA constructs targeting ISG20L2 were generated, with the following target sequences: sh-ISG20L2-1, GATGTAGCATTGTCAACTACA; sh-ISG20L2-2, GCTGCAGTGTCTTGGTTGACC; and sh-ISG20L2-3, AGCTCATTCAGAGAATAAATG. A GFP-labeled non-targeting plasmid was used as the negative control. Correct insertion of shRNA sequences was confirmed by Sanger sequencing. AGS and HGC-27 cells were seeded into 6-well plates at 5 × 10^5^ cells/well and transfected at 50%–60% confluence with 2 μg plasmid and 5 μL Lipofectamine 3000 (Beyotime, China) per well according to the manufacturer’s instructions. After 6 h, the medium was replaced with fresh complete medium, and cells were cultured for an additional 48 h. Transfection efficiency was evaluated by GFP fluorescence using an inverted fluorescence microscope (Olympus IX73, Japan). Knockdown efficiency of the three constructs was then assessed by quantitative real-time PCR (qRT-PCR) and western blotting, and the two most effective shRNAs were selected for subsequent experiments.

### 2.10 Quantitative real-time PCR

Total RNA was extracted from transfected cells using standard procedures, followed by reverse transcription to synthesize complementary DNA (cDNA). qRT-PCR was then performed to detect ISG20L2 mRNA expression in the control and knockdown groups. The qRT-PCR amplification was conducted using SYBR qPCR Master Mix (Beyotime, Shanghai, China). Relative ISG20L2 expression was normalized to Tubulin and calculated using the 2−ΔΔCt method. The primer sequences were as follows: ISG20L2 forward, 5′-AGAAGCTGCTAAACCGGGATATC-3′; ISG20L2 reverse, 5′-CCCACTCGACTTCAACCAACTTA-3′; Tubulin forward, 5′-CTGGACCGCATCTCTGTGTACTA-3′; and Tubulin reverse, 5′-GTACCACATCCAGGACAGAATCA-3′.

### 2.11 Cell proliferation, colony formation, wound-healing, Transwell migration, and invasion assays

The effects of ISG20L2 knockdown on the proliferative, clonogenic, migratory and invasive abilities of gastric cancer cells were evaluated using CCK-8, colony formation, wound-healing and Transwell assays. AGS and HGC-27 cells transfected with sh-NC were used as negative controls, whereas cells transfected with sh-ISG20L2–2 or sh-ISG20L2–3 were used as ISG20L2 knockdown groups. Functional assays were performed 48 h after plasmid transfection, and each experiment was conducted with three replicate wells per group. Cell proliferation was assessed using a Cell Counting Kit-8 assay (CCK-8; Solarbio, China). Briefly, transfected AGS and HGC-27 cells were digested with 0.25% trypsin-EDTA, resuspended in complete medium, and seeded into 96-well plates at a density of 5 × 10^4^ cells/well in 100 μL medium. Blank wells containing medium without cells were included as background controls. At days 1, 2, 3, 4 and 5 after seeding, 10 μL of CCK-8 reagent was added to each well, followed by incubation at 37 °C for 2 h. The absorbance at 450 nm was measured using a microplate reader (Thermo Scientific, USA), and cell proliferation curves were generated based on the optical density values. For the colony formation assay, transfected cells were seeded into 6-well plates at a density of 5 × 10^2^ cells/well and cultured in complete medium for 14 days. The medium was replaced every 3 days. At the end of the incubation period, colonies were washed twice with PBS, fixed with 4% paraformaldehyde for 30 min, and stained with crystal violet solution (Solarbio, China) for 15 min. After washing and air-drying, colonies were photographed and counted. Colonies with a diameter of ≥50 μm were included in the analysis. For the wound-healing assay, transfected AGS and HGC-27 cells were seeded into 6-well plates at a density of 5 × 10^5^ cells/well and cultured until the cell monolayer reached more than 90% confluence. A linear wound was generated using a sterile 200-μL pipette tip. Detached cells were removed by washing twice with PBS, and the cells were subsequently cultured in serum-free DMEM. Images of the wound area were captured at 0 h and 48 h using an inverted microscope (Olympus IX73, Japan) at ×100 magnification. Wound width was measured using ImageJ software, and the migration rate was calculated as follows: migration rate (%) = [(wound width at 0 h − wound width at 48 h)/wound width at 0 h] × 100%. Cell migration and invasion were further evaluated using Transwell chambers with 8-μm pore membranes (Corning, USA). For the migration assay, transfected cells were suspended in serum-free DMEM and seeded into the upper chamber at a density of 2 × 10^4^ cells in 200 μL medium. The lower chamber was filled with 600 μL DMEM containing 10% fetal bovine serum as a chemoattractant. After incubation at 37 °C for 48 h, non-migrated cells on the upper surface of the membrane were gently removed with cotton swabs. Cells that had migrated to the lower surface were fixed with 4% paraformaldehyde for 30 min, stained with crystal violet for 15 min, washed, air-dried and photographed under an inverted microscope at ×200 magnification. Five random microscopic fields were selected for each group, and the average number of migrated cells was calculated. For the invasion assay, the upper chambers were pre-coated with Matrigel matrix (BD Biosciences, USA). Matrigel was diluted with serum-free DMEM at a ratio of 1:8, and 50 μL of diluted Matrigel was added to the upper surface of each membrane. The chambers were incubated at 37 °C for 4 h to allow gel solidification. The subsequent procedures, including cell seeding, incubation, fixation, staining, imaging and quantification, were performed as described for the migration assay. The number of cells that invaded through the Matrigel-coated membrane was used to evaluate invasive capacity.

### 2.12 Statistical analysis

All statistical analyses were performed using R software version 4.3.2 and GraphPad Prism version 9.0. Bioinformatics analyses based on public databases, including TCGA, GTEx, CancerSEA, MeTIL and TIP, were conducted in R, whereas experimental validation data were analyzed using GraphPad Prism. Data are presented as mean ± standard deviation (SD), unless otherwise indicated. Differences between two independent groups were analyzed using Student’s t-test or the Wilcoxon rank-sum test according to data distribution. Paired comparisons between gastric cancer tissues and matched adjacent non-tumor tissues were performed using paired Student’s t-test or the Wilcoxon matched-pairs signed-rank test. Comparisons among more than two groups, including MSI subtypes and cell-cycle phases, were conducted using one-way ANOVA or the Kruskal–Wallis test, as appropriate. Correlations between ISG20L2 expression and promoter methylation, functional state scores, signaling pathway activity, immune infiltration, immunoregulatory features, cell-cycle activity and selected gene expression were assessed using Pearson or Spearman correlation analysis according to data characteristics. For *in vitro* experiments, quantitative data from qRT-PCR, western blotting, CCK-8, colony formation, wound-healing and Transwell assays were obtained from at least three independent experiments unless otherwise specified. A value of P < 0.05 was considered statistically significant, and P < 0.01 was considered highly significant. Statistical significance was indicated as ns, not significant (P ≥ 0.05); *P < 0.05; **P < 0.01; ***P < 0.001; ****P < 0.0001.

## 3 Results

### 3.1 ISG20L2 is aberrantly upregulated across multiple cancers and in gastric cancer

To characterize the expression pattern of ISG20L2 in human malignancies, we first performed a pan-cancer analysis using integrated TCGA and GTEx datasets. As shown in **Figure 1A**, ISG20L2 was dysregulated across multiple tumor types and displayed an overall upregulated trend in liver, gastric, intestinal and brain tumors compared with corresponding normal tissues. To provide complementary validation of this pan-cancer expression pattern, TCGA-only analysis showed significant differences in ISG20L2 expression between tumor and normal tissues in multiple cancers (**Figure 1B**), while integrated TCGA–GTEx analysis after tissue-specific batch correction further confirmed this dysregulated expression pattern (**Figure 1C**). In gastric cancer, paired analysis further demonstrated that ISG20L2 expression was significantly higher in STAD tissues than in matched adjacent non-tumor tissues (**Figure 1D**, P < 0.05). In addition, ISG20L2 expression differed significantly among microsatellite instability (MSI) subtypes (**Figure 1E**) and was negatively correlated with promoter methylation levels (**Figure 1F**). Collectively, these findings indicate that ISG20L2 is aberrantly overexpressed in gastric cancer and may be associated with specific molecular and epigenetic features.

**Figure 1:**
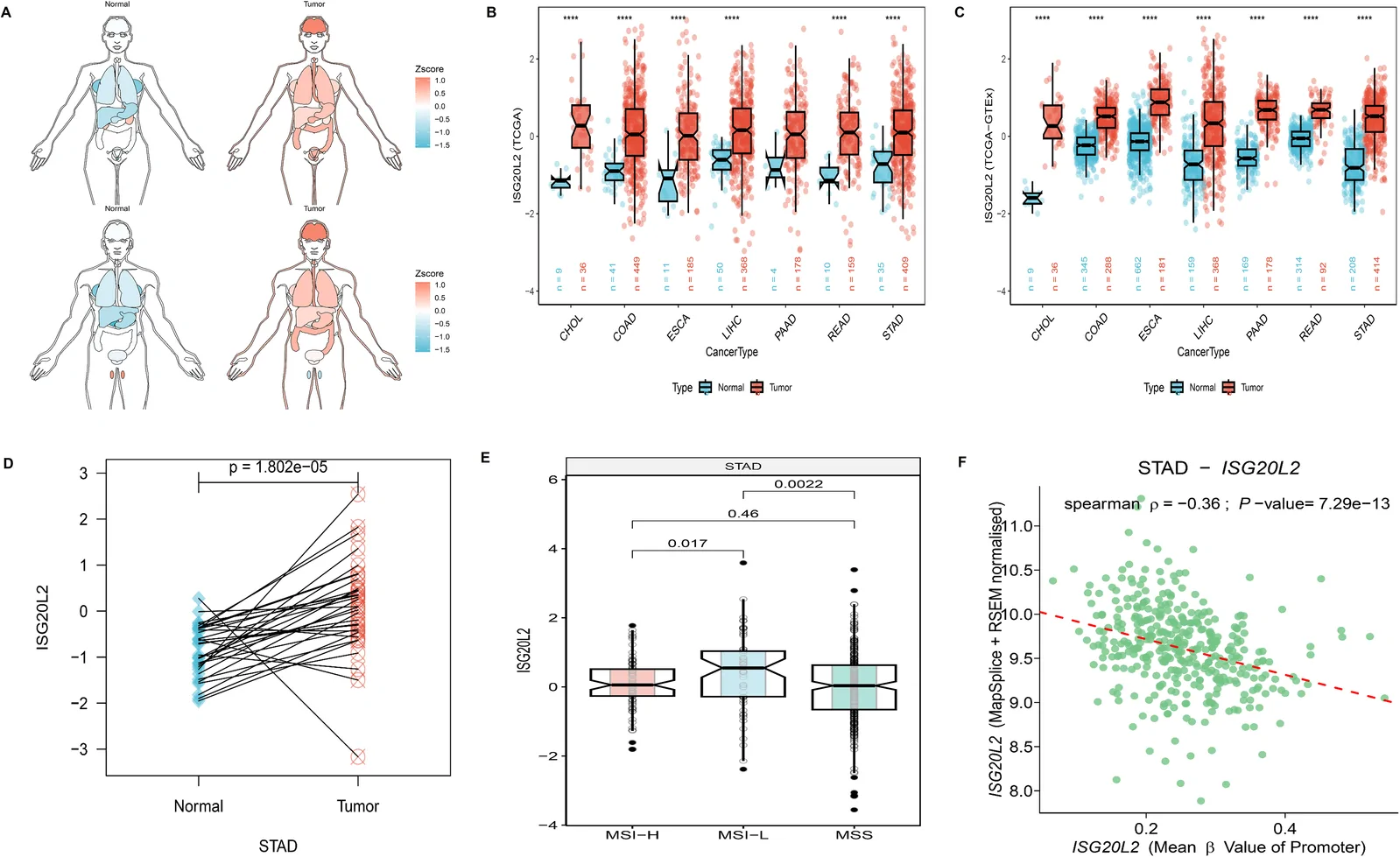
Pan-cancer expression profile of ISG20L2 and its association with molecular features in gastric cancer. **(A)** Integrated analysis of the TCGA and GTEx datasets showed the overall pan-cancer expression pattern of ISG20L2, with predominant upregulation observed in liver, gastric, intestinal and brain tumors relative to corresponding normal tissues. **(B)** As a complementary analysis, TCGA-only data demonstrated significant differences in ISG20L2 expression between tumor and adjacent non-tumor tissues across multiple cancer types. **(C)** Integrated TCGA–GTEx analysis after tissue-specific correction of TCGA-versus-GTEx source effects further confirmed the differential expression pattern of ISG20L2 across multiple cancer types. **(D)** Paired analysis in STAD revealed that ISG20L2 expression was significantly higher in tumor tissues than in matched adjacent non-tumor tissues, with paired samples from the same patient connected by lines. **(E)** Microsatellite instability (MSI)-based stratification analysis revealed significant differences in ISG20L2 expression among distinct MSI subtypes, indicating an association between ISG20L2 and the molecular heterogeneity of gastric cancer. **(F)** Correlation analysis showed that ISG20L2 expression was inversely associated with promoter methylation levels, indicating that promoter hypomethylation may contribute to its aberrant upregulation in gastric cancer. Statistical significance was indicated as ****P < 0.0001.

### 3.2 ISG20L2 is preferentially expressed in exhausted CD8+ T cells at the single-cell level

To further define the cellular distribution of ISG20L2 in gastric cancer, we analyzed two independent single-cell RNA-sequencing datasets, STAD_GSE134520 and STAD_GSE167297. After normalization, batch correction and cell-type annotation, UMAP analysis delineated the cellular composition of the gastric tumor microenvironment in both cohorts (**Figures 2A**, **D**). Cell type-specific expression profiling revealed heterogeneous ISG20L2 expression across annotated cell populations (**Figures 2B**, **E**). Notably, feature plots showed that ISG20L2 was preferentially expressed in the CD8Tex in both datasets, with the highest average expression observed in this population (**Figures 2C**, **F**). These results indicate that ISG20L2 is enriched in exhausted CD8+ T cells and may be associated with T-cell dysfunction and an immunosuppressive tumor microenvironment in gastric cancer.

**Figure 2:**
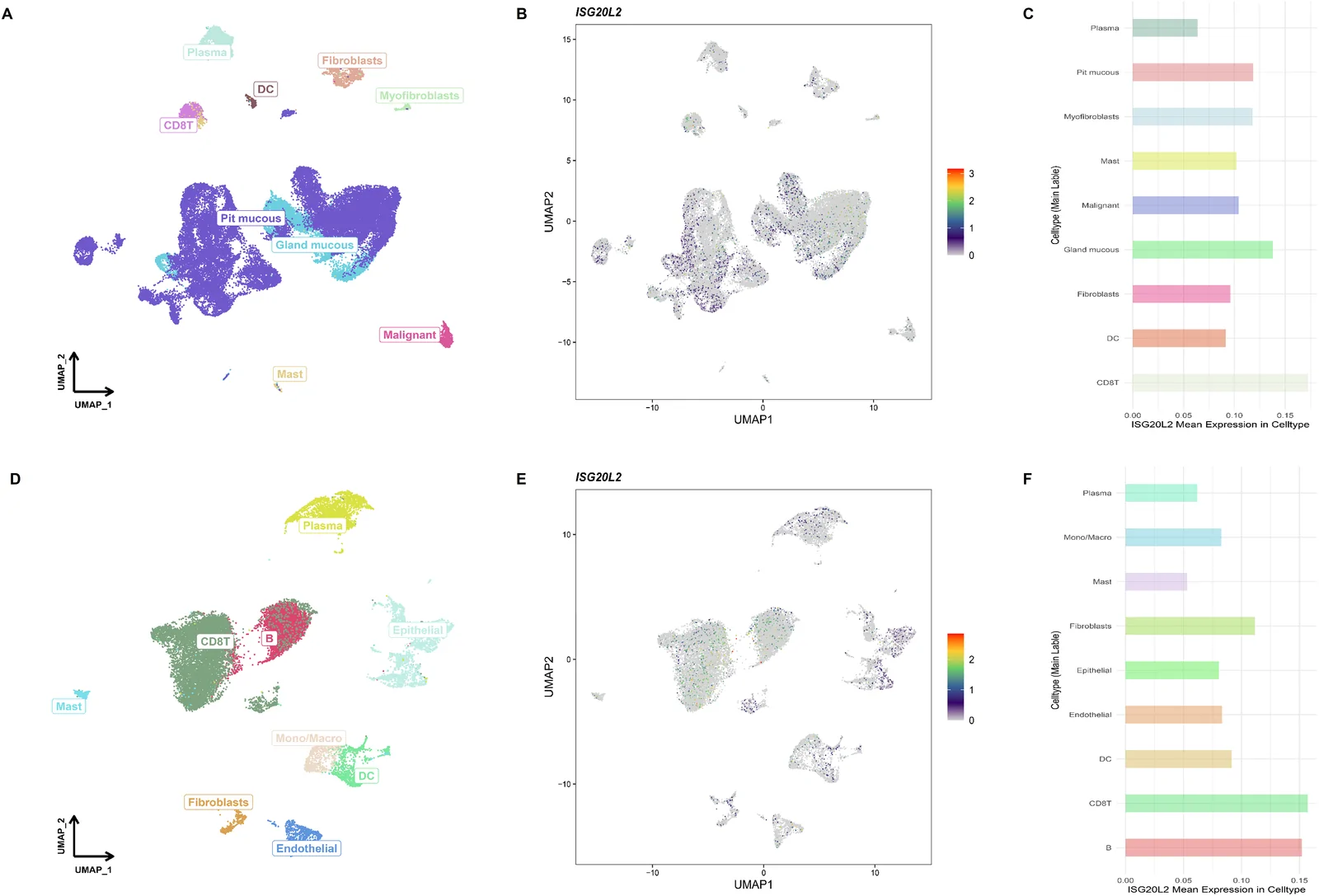
Single-cell transcriptomic analysis reveals preferential expression of ISG20L2 in exhausted CD8+ T cells in gastric cancer. **(A)** UMAP visualization of single-cell RNA-sequencing data from the STAD_GSE134520 cohort showing the distribution of major cell populations in the gastric cancer microenvironment after normalization, batch-effect correction and cell-type annotation. **(B)** Cell type-specific expression analysis of ISG20L2 in the STAD_GSE134520 cohort, showing differential expression across annotated cell populations. **(C)** Feature plot of ISG20L2 expression in the STAD_GSE134520 dataset, demonstrating that ISG20L2 is predominantly enriched in the CD8Tex, in which the highest expression level was observed. **(D)** UMAP visualization of single-cell RNA-sequencing data from the independent STAD_GSE167297 cohort showing the overall distribution of annotated cell clusters after normalization, batch correction and dimensionality reduction. **(E)** Cell type-specific expression analysis of ISG20L2 in the STAD_GSE167297 cohort, confirming variable expression across different cell populations. **(F)** Feature plot of ISG20L2 expression in the STAD_GSE167297 dataset, further confirming its preferential expression in the CD8Tex subset, consistent with the findings from the STAD_GSE134520 cohort. Together, these results indicate that ISG20L2 is preferentially enriched in exhausted CD8^+^ T cells in gastric cancer, suggesting a potential association with the exhausted T-cell state; however, this expression pattern alone does not establish a direct role for ISG20L2 in promoting T-cell exhaustion. Statistical significance is indicated as P < 0.05 and P < 0.01.

### 3.3 ISG20L2 is positively associated with malignant functional states in gastric cancer

To explore the biological significance of ISG20L2 in gastric cancer, we next assessed its association with tumor functional states using the CancerSEA database. Pearson correlation analysis showed that ISG20L2 expression was positively associated with multiple malignant phenotypes, including angiogenesis, apoptosis, differentiation, epithelial-mesenchymal transition, hypoxia, inflammation, invasion, proliferation and stemness (**Figure 3A**). Moreover, in STAD, ISG20L2 expression was significantly positively correlated with the cell-cycle functional state score (**Figure 3B**, P < 0.05). Together, these data suggest that elevated ISG20L2 expression is closely linked to malignant biological programs in gastric cancer.

**Figure 3:**
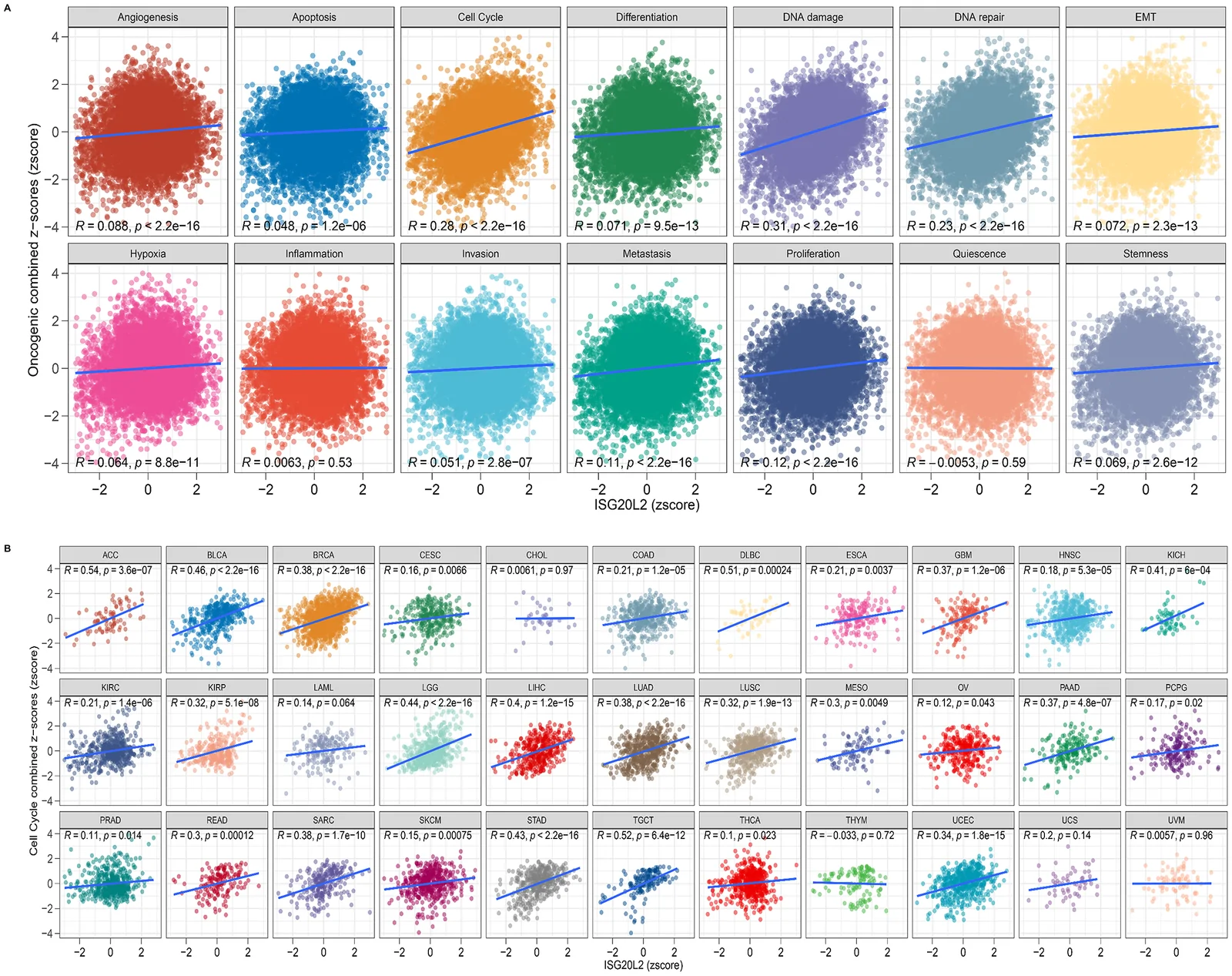
Functional state analysis reveals a positive association between ISG20L2 expression and malignant phenotypes in gastric cancer. **(A)** Pearson correlation analysis based on the CancerSEA database showed that ISG20L2 expression was positively associated with multiple tumor-related functional states in gastric cancer, including angiogenesis, apoptosis, differentiation, EMT, hypoxia, inflammation, invasion, proliferation and stemness. **(B)** In STAD, ISG20L2 expression was significantly positively correlated with the cell-cycle functional state score, suggesting a potential link between ISG20L2 and enhanced proliferative activity. Statistical significance is indicated as P < 0.05 and P < 0.01.

### 3.4 ISG20L2 is associated with distinct signaling pathway activities in gastric cancer

Given the association between ISG20L2 and malignant phenotypes, we further investigated its relationship with tumor-related signaling pathways across cancers. Significant differences in several signaling pathway activity scores were observed between the ISG20L2-high and ISG20L2-low groups in STAD (**Figures 4A–I**). Among the pathways examined, PI3K (P = 2.161 × 10^−6^), TGFβ (P = 3.160 × 10^−6^), MAPK (P = 5.152 × 10^−5^), TRAIL (P = 1.348 × 10^−4^), and JAK–STAT (P = 0.009) showed the most significant differences. PI3K and MAPK pathway activities were higher in the ISG20L2-high group, whereas TGFβ, TRAIL, and JAK–STAT pathway activities were lower. These findings identify several signaling pathways most strongly associated with ISG20L2 expression status in STAD and suggest that elevated ISG20L2 expression is accompanied by distinct alterations in tumor-related signaling programs.

**Figure 4:**
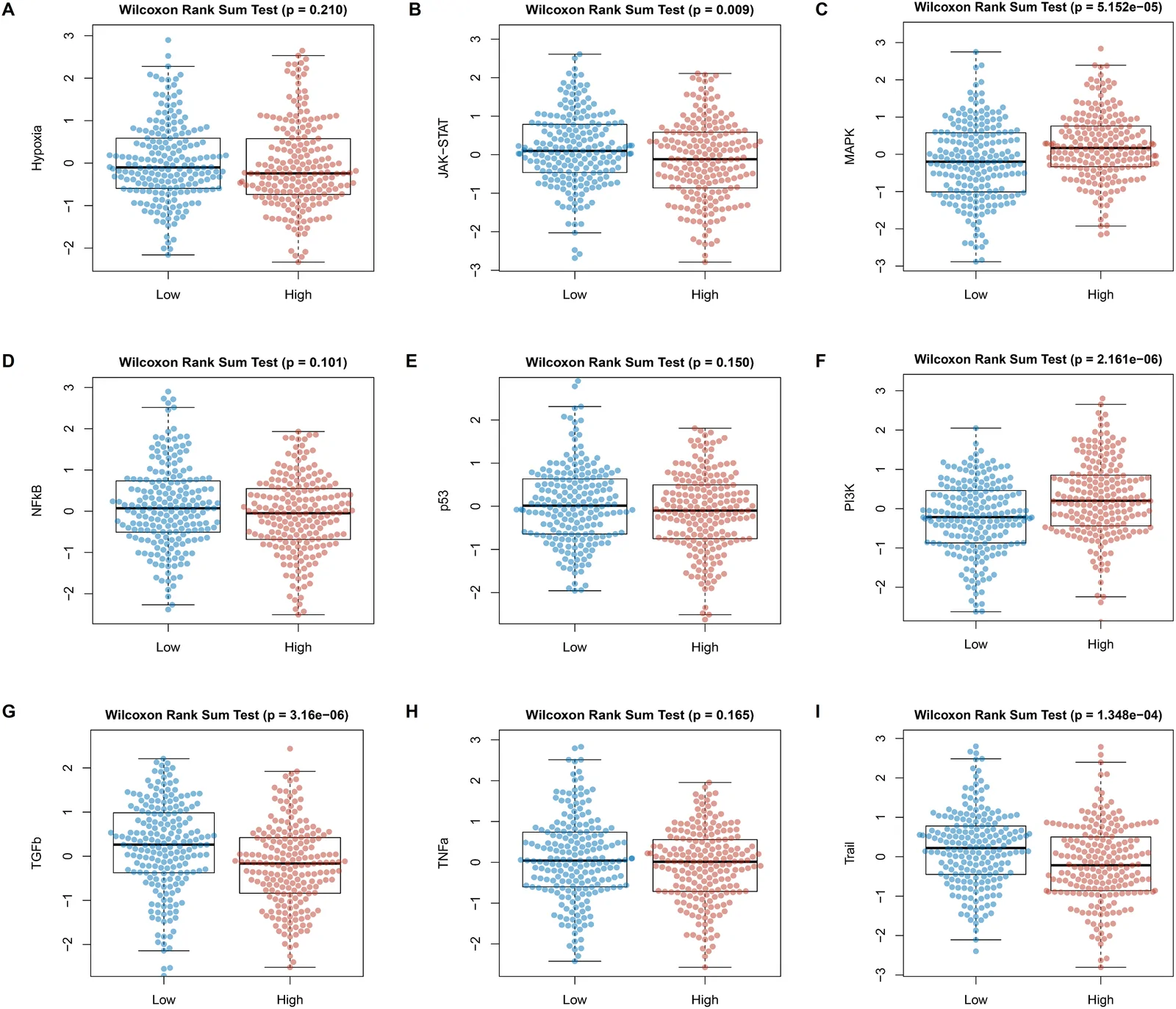
Correlation analysis of ISG20L2-associated signaling pathways across cancers reveals prominent enrichment in gastric cancer. **(A–I)** Correlation analysis showed that ISG20L2 was associated with multiple cancer-related signaling pathways across common tumor types, with the most significant enrichment observed in STAD. Statistical significance is indicated as P < 0.05 and P < 0.01.

### 3.5 ISG20L2 is associated with activation of cell-cycle-related programs in gastric cancer

To identify the dominant biological programs associated with ISG20L2 expression, pathway enrichment analyses were performed using STAD transcriptomic data. Although ISG20L2 expression showed associations with multiple tumor-related pathways, cell-cycle-associated programs represented the most prominent enrichment signals in the ISG20L2-high group (**Figures 5A–C**). Gene set enrichment analysis demonstrated that genes positively associated with ISG20L2 expression were significantly enriched in pathways related to DNA replication, mitotic progression and cell-cycle regulation, suggesting that ISG20L2 may participate in proliferative regulation. Consistently, ISG20L2 expression exhibited a significant positive correlation with cell-cycle activity scores in STAD (**Figure 5D**). Furthermore, ISG20L2 expression varied across different cell-cycle phases, with increased expression observed in proliferative cell-cycle states (**Figures 5E, F**). Collectively, these findings identify cell-cycle activation as a major biological feature associated with ISG20L2 expression in gastric cancer.

**Figure 5:**
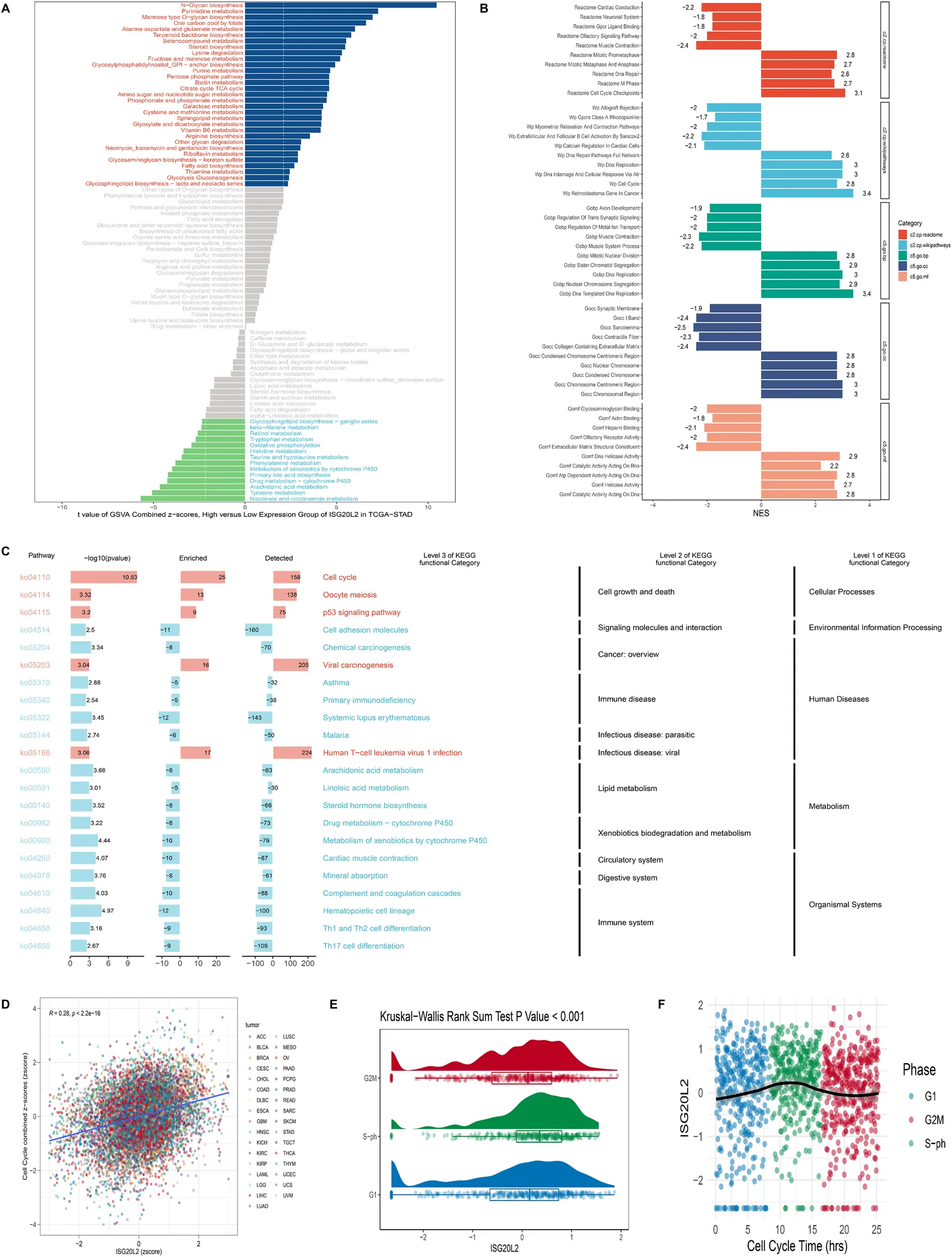
ISG20L2 is associated with activation of cell-cycle-related programs in gastric cancer. **(A, B)** Pathway enrichment analyses identified cell-cycle-related pathways as the predominant biological programs associated with ISG20L2 expression in STAD. **(C)** GSEA demonstrated preferential enrichment of cell-cycle-associated genes in the ISG20L2-high group, whereas genes downregulated in the ISG20L2-high group showed enrichment in other biological processes. **(D)** Correlation analysis showed a positive association between ISG20L2 expression and cell-cycle activity scores. **(E, F)** ISG20L2 expression differed significantly among different cell-cycle phases, suggesting a relationship between ISG20L2 expression and proliferative states. Statistical significance is indicated as P < 0.05 and P < 0.01.

### 3.6 ISG20L2 is associated with immune infiltration and antitumor immune activity in gastric cancer

Given the preferential expression of ISG20L2 in exhausted CD8^+^ T cells identified by single-cell RNA sequencing, we next investigated whether ISG20L2 was associated with the broader immune microenvironment in gastric cancer. Multi-algorithm immune deconvolution analysis revealed significant associations between ISG20L2 expression and multiple infiltrating immune-cell populations in STAD (**Figure 6A**). We next applied the MeTIL approach to further compare the estimated infiltration levels of major immune-cell populations between the ISG20L2-high and ISG20L2-low groups. Differences were observed in the estimated infiltration of T cells, NK cells, B cells and regulatory T cells between the two groups (**Figure 6B**). In addition, Tumor Immunophenotype Profiling (TIP) analysis demonstrated significant associations between ISG20L2 expression and several steps of the antitumor immune response, including immune-cell recruitment and tumor-cell killing-related processes (**Figure 6C**). Collectively, these findings indicate that ISG20L2 expression is associated with alterations in the immune-cell landscape and antitumor immune activity in gastric cancer.

**Figure 6:**
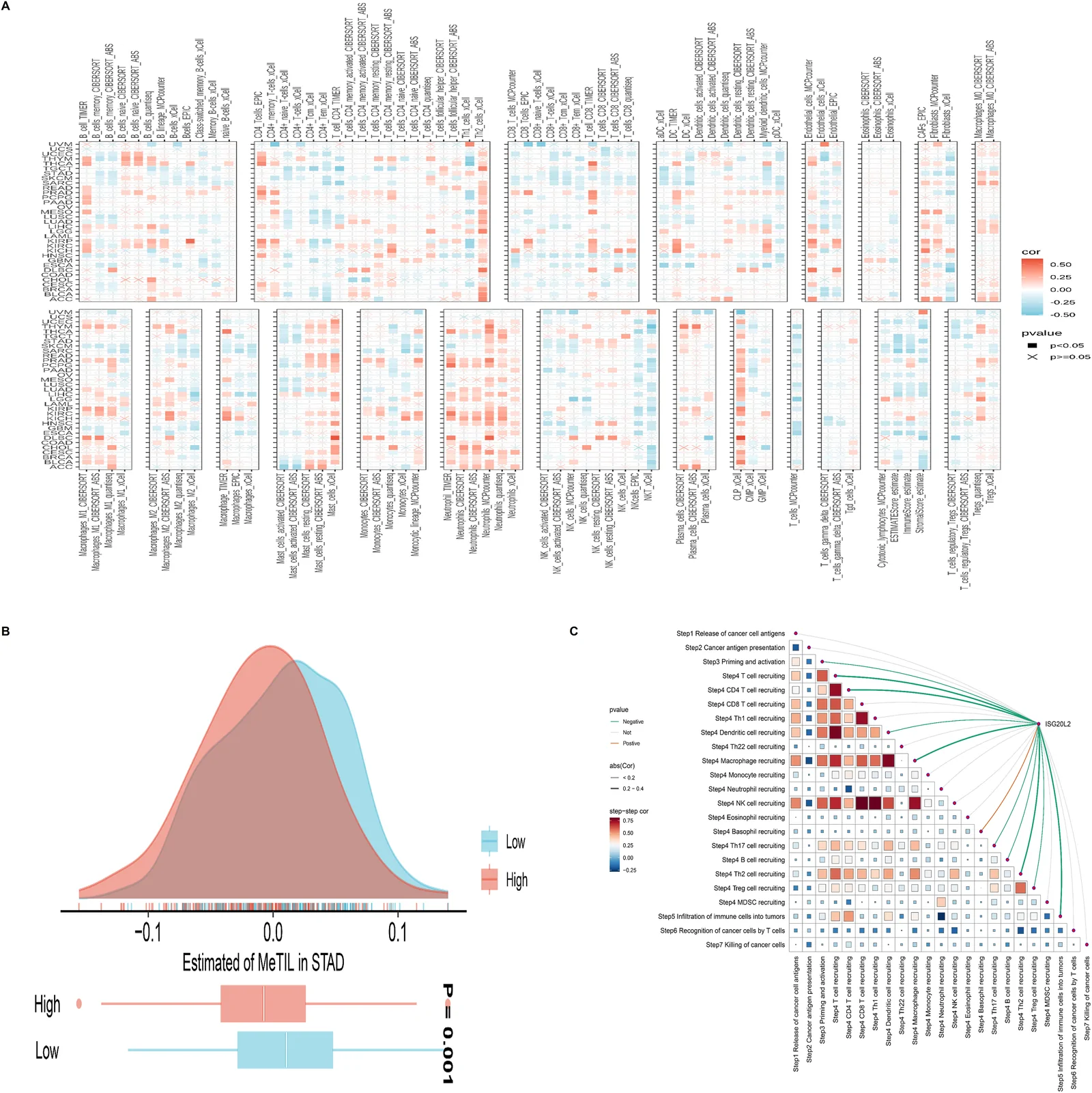
ISG20L2 is associated with immune infiltration and antitumor immune activity in gastric cancer. **(A)** Spearman correlation analysis using multiple immune deconvolution algorithms showing the associations between ISG20L2 expression and infiltrating immune-cell populations in gastric cancer. **(B)** MeTIL analysis comparing the estimated infiltration levels of T cells, NK cells, B cells and regulatory T cells between the ISG20L2-high and ISG20L2-low groups. **(C)** Tumor Immunophenotype Profiling (TIP) analysis showing the correlations between ISG20L2 expression and antitumor immune-response processes. Statistical significance is indicated as P < 0.05 and P < 0.01.

### 3.7 ISG20L2 is associated with immunoregulatory features and shows variable relationships with CD8^+^ T-cell exhaustion markers

We next examined whether ISG20L2 expression was associated with specific immunoregulatory characteristics in gastric cancer. Correlation analysis across immune-related features showed that ISG20L2 was associated with several immune-regulatory parameters, including the ratio of proliferative to activated T cells and PD-L1 expression in STAD (**Figure 7A**). These findings suggested that ISG20L2 may be linked not only to immune-cell infiltration but also to the functional state of the tumor immune microenvironment. Because single-cell analysis demonstrated preferential ISG20L2 expression in exhausted CD8^+^ T cells, we further investigated the relationship between ISG20L2 and representative CD8^+^ T-cell exhaustion-associated genes, including PDCD1, LAG3 and TIGIT (**Figures 7B–D**). In the current analysis, the associations were not uniformly positive across the three markers. ISG20L2 showed no significant correlation with PDCD1 or TIGIT expression, whereas a modest negative association was observed with LAG3 expression. These findings suggest that the relationship between ISG20L2 and individual exhaustion-associated genes may be heterogeneous at the bulk-tumor level. Nevertheless, together with the single-cell finding that ISG20L2 is preferentially expressed in exhausted CD8^+^ T cells, these data support an association between ISG20L2 and the immune-regulatory landscape of gastric cancer, while indicating that its relationship with individual exhaustion markers requires further investigation.

**Figure 7:**
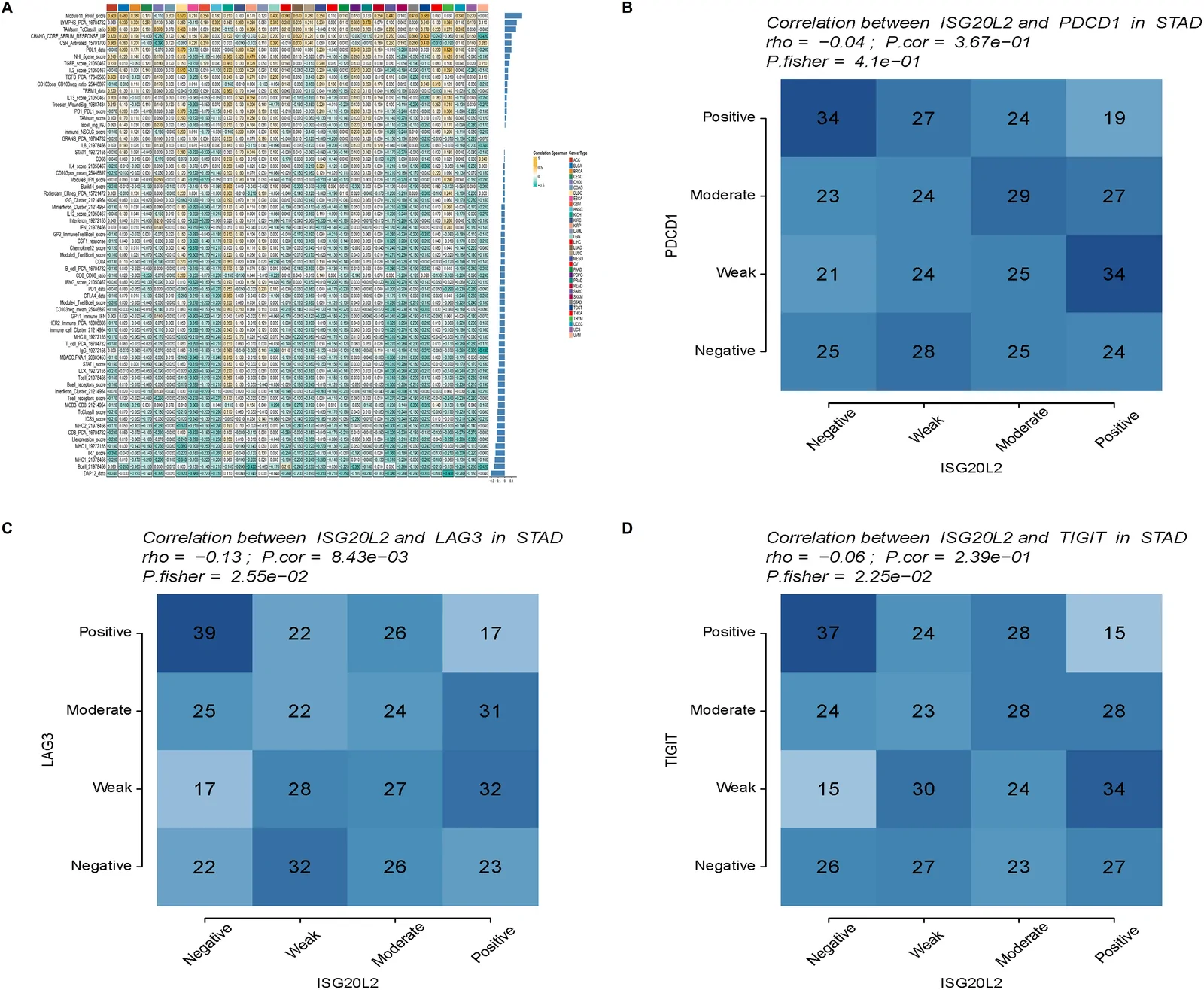
ISG20L2 is associated with immunoregulatory features and shows variable relationships with CD8^+^ T-cell exhaustion markers. **(A)** Heatmap illustrating the associations between ISG20L2 expression and immune-related characteristics across cancers, highlighting the relationships with the proliferative/activated T-cell ratio and PD-L1 expression in STAD. **(B–D)** Distribution analyses illustrating the association between ISG20L2 expression levels and CD8^+^ T-cell exhaustion-related markers, including PDCD1 **(B)**, LAG3 **(C)**, and TIGIT **(D)**. * indicates P < 0.05, **** indicates P < 0.0001.

### 3.8 ISG20L2 is associated with genomic alteration patterns and immunotherapy-related characteristics

To further explore the genomic and potential therapeutic relevance of ISG20L2, we examined mutation patterns, virtual knockout-associated transcriptional alterations and computationally inferred immunotherapy-related characteristics. Mutation landscape analysis revealed the genomic alteration profile associated with ISG20L2 expression in gastric cancer (**Figure 8A**). Virtual knockout analysis based on TCGA transcriptomic data suggested that ISG20L2 depletion was associated with substantial gene-expression alterations (**Figures 8B, C**). Correlation analyses of selected genes further identified significant associations with ISG20L2 expression (**Figures 8D–F**). Computational immunotherapy-related analyses suggested that ISG20L2 expression was associated with differential immunotherapy-related characteristics (**Figures 8G–I**). These findings indicate that ISG20L2 is associated with genomic alteration patterns, downstream transcriptional changes and predicted immune-related therapeutic features in gastric cancer. To further evaluate the potential prognostic relevance of ISG20L2, we examined its association with multiple survival endpoints in publicly available gastric cancer datasets (**Supplementary Figure 1**). When patients were stratified into quartiles according to ISG20L2 expression, no significant differences were observed in DFI, DSS, OS, or PFI (log-rank P = 0.681, 0.528, 0.329, and 0.369, respectively; **Supplementary Figures 1A–D**). Using optimized expression cutoffs, differences in DFI, DSS, and PFI also did not reach statistical significance (P = 0.063, 0.067, and 0.102, respectively; **Supplementary Figures 1E, F, H**). Although an optimized cutoff identified a nominal association with OS (P = 0.044; **Supplementary Figure 1G**), this finding was not reproduced using quartile-based stratification. Collectively, these analyses did not demonstrate a consistent association between ISG20L2 expression and survival outcomes, suggesting that its prognostic significance in gastric cancer remains uncertain.

**Figure 8:**
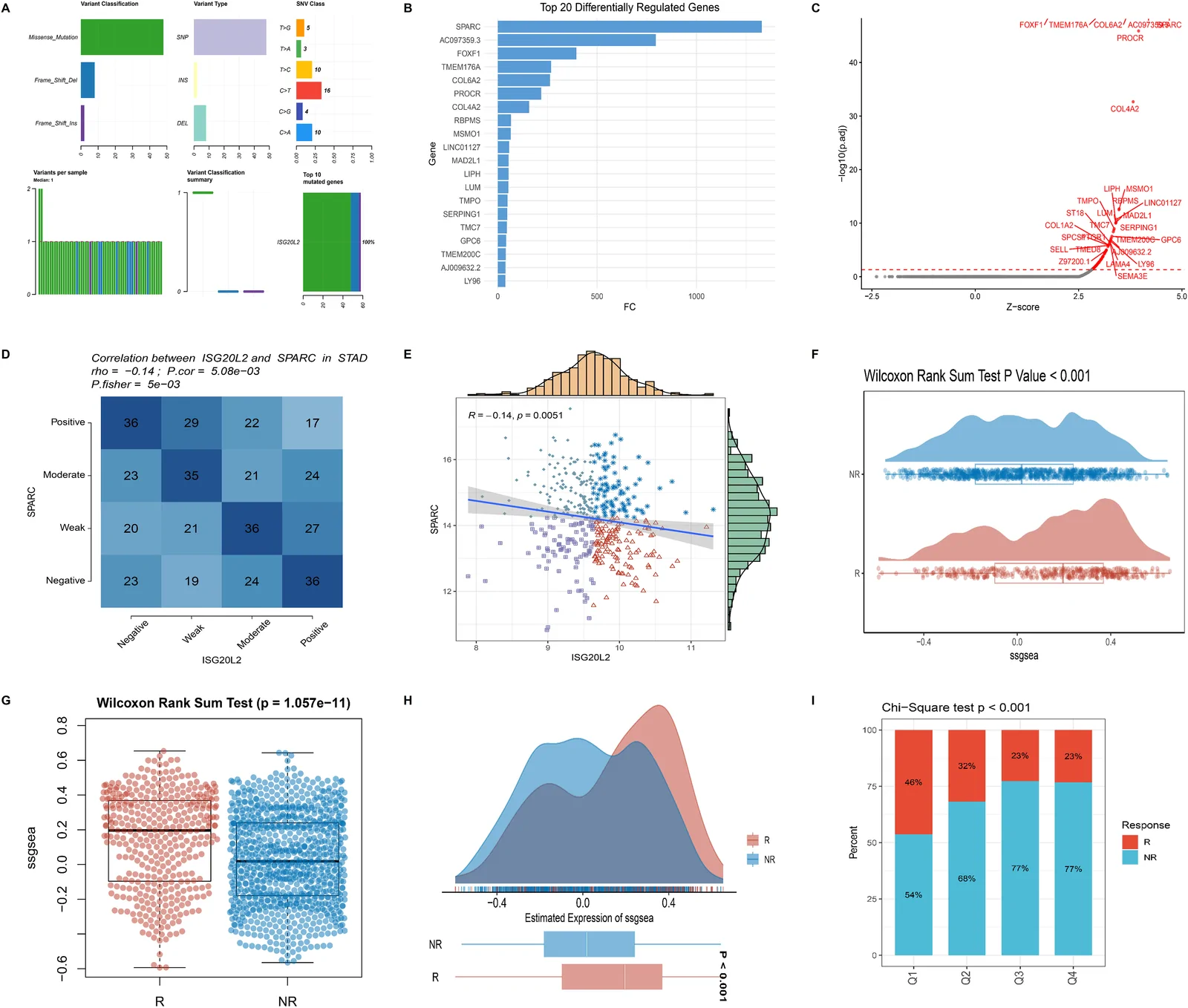
Genomic alteration, virtual knockout and immunotherapy-related analyses of ISG20L2 in gastric cancer. **(A)** Mutation landscape analysis showing genomic alteration patterns associated with ISG20L2 expression in gastric cancer. **(B, C)** Virtual knockout analysis based on TCGA transcriptomic data showing predicted gene-expression alterations associated with simulated ISG20L2 depletion. **(D–F)** Correlation analyses of selected genes showing significant associations with ISG20L2 expression. **(G–I)** Computational immunotherapy-related analyses showing differences in predicted immunotherapy-associated characteristics according to ISG20L2 expression. Statistical significance is indicated as P < 0.05 and P < 0.01. ** indicates P < 0.01, **** indicates P < 0.0001.

### 3.9 ISG20L2 is upregulated at the protein level in gastric cancer cells and tissues

To validate the abnormal expression of ISG20L2 at the protein level, western blotting was performed in gastric cancer cell lines and clinical samples. Compared with the normal gastric epithelial cell line GES-1, gastric cancer cell lines exhibited significantly higher ISG20L2 protein expression (**Figure 9A**). Moreover, analysis of 9 paired STAD tissues and adjacent non-tumor tissues further confirmed that ISG20L2 protein levels were elevated in tumor tissues (**Figure 9B**). These findings provide protein-level evidence that ISG20L2 is upregulated in gastric cancer.

**Figure 9:**
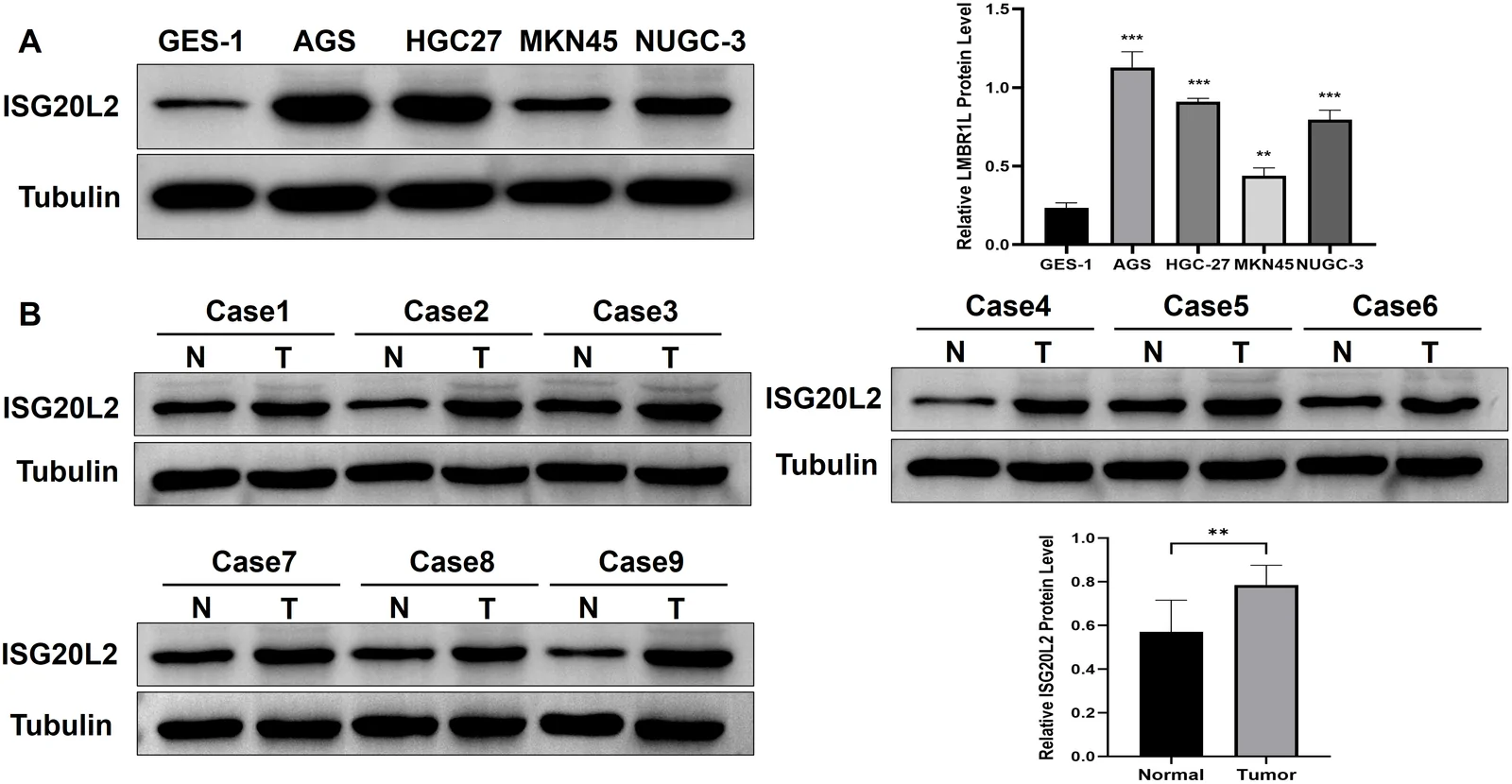
ISG20L2 is upregulated at the protein level in gastric cancer. **(A)** Western blot analysis showed that ISG20L2 protein expression was significantly higher in gastric cancer cell lines than in the normal gastric epithelial cell line GES-1. **(B)** Western blot analysis of 9 paired STAD and adjacent non-tumor tissues further confirmed that ISG20L2 protein expression was elevated in tumor tissues. Statistical significance was indicated as ns, not significant (P ≥ 0.05); **P < 0.01; and ***P < 0.001.

### 3.10 ISG20L2 knockdown is efficiently established in AGS and HGC-27 cells

To investigate the biological function of ISG20L2 in gastric cancer cells, we established ISG20L2 knockdown models in AGS and HGC-27 cells and first evaluated silencing efficiency. Fluorescence microscopy was used to assess transfection efficiency (**Figure 10A**). Quantitative PCR showed that ISG20L2 mRNA expression was significantly reduced in the knockdown group compared with the control group (**Figure 10B**), and western blot analysis further confirmed efficient depletion of ISG20L2 at the protein level (**Figure 10C**). These results demonstrate the successful establishment of effective ISG20L2 knockdown models in both gastric cancer cell lines.

**Figure 10:**
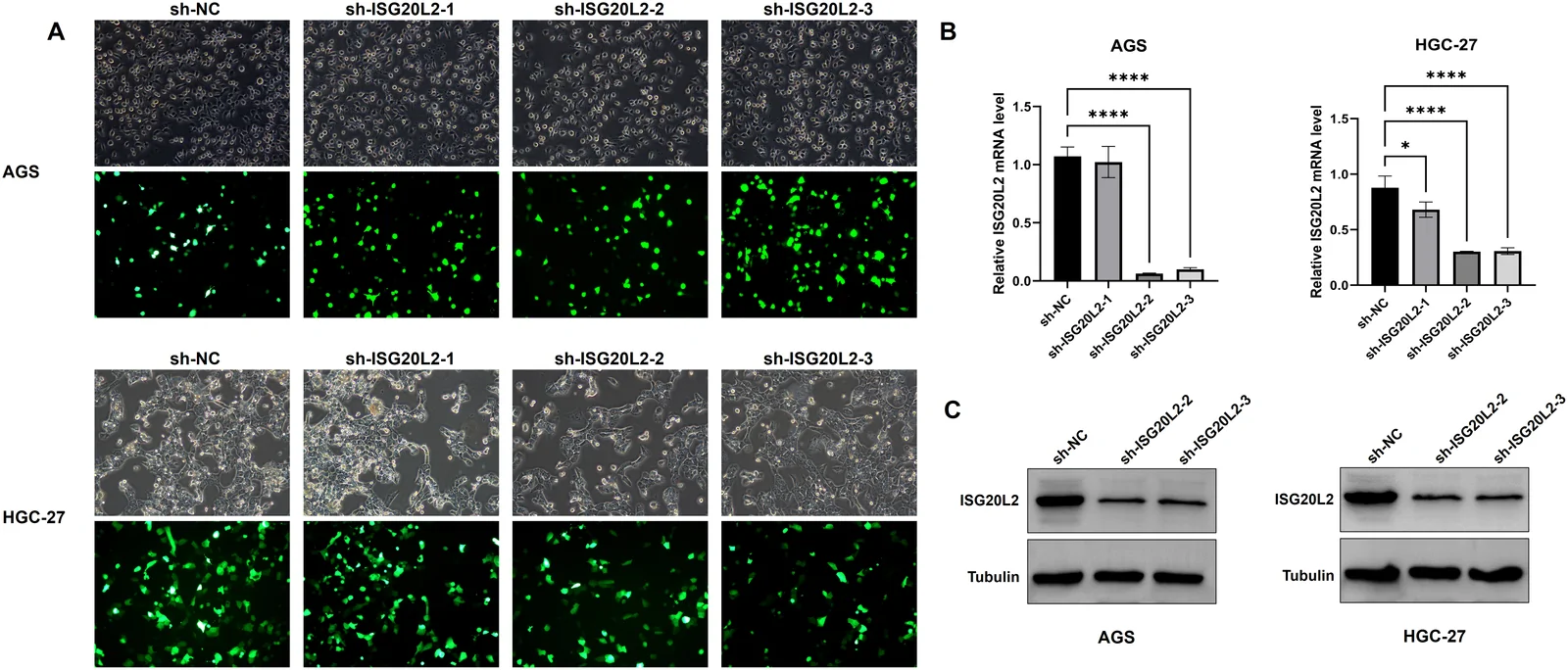
Validation of ISG20L2 knockdown efficiency in AGS and HGC-27 cells. **(A)** Transfection efficiency in ISG20L2 knockdown AGS and HGC-27 cells was assessed by fluorescence microscopy. **(B)** Quantitative PCR analysis confirmed that ISG20L2 mRNA expression was significantly reduced in the knockdown group compared with the control group. **(C)** Western blot analysis further verified efficient ISG20L2 knockdown at the protein level. Statistical significance was indicated as ns, not significant (P ≥ 0.05); *P < 0.05; and ****P < 0.0001.

### 3.11 ISG20L2 knockdown suppresses the proliferative, migratory and invasive phenotypes of gastric cancer cells

To determine whether ISG20L2 contributes to malignant cellular behavior, we next assessed proliferation, clonogenicity, migration and invasion after gene silencing in AGS and HGC-27 cells. CCK-8 assays showed that ISG20L2 knockdown significantly reduced cell viability and proliferative capacity compared with the control group (**Figure 11A**), while colony formation assays demonstrated a marked decrease in the colony-forming ability of both cell lines following ISG20L2 depletion (**Figure 11B**). In parallel, wound-healing assays showed that ISG20L2 silencing significantly impaired the migratory capacity of AGS and HGC-27 cells (**Figures 12A, B**). Consistently, Transwell assays further demonstrated that depletion of ISG20L2 markedly inhibited both migration and invasion in these two cell lines (**Figures 12C, D**). Taken together, these *in vitro* findings support a role for ISG20L2 in maintaining malignant phenotypes of gastric cancer cells.

**Figure 11:**
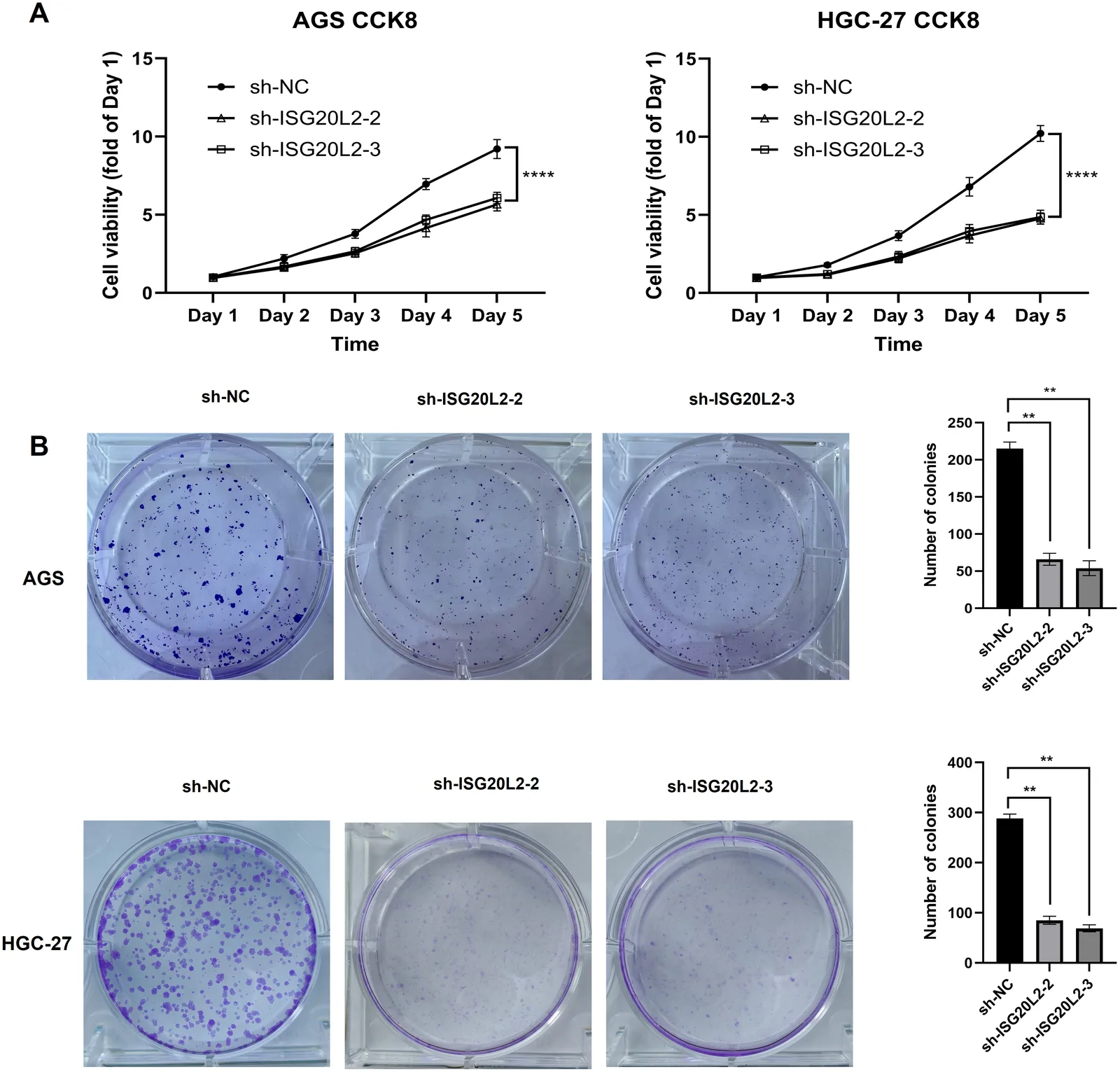
ISG20L2 knockdown suppresses the proliferative capacity of AGS and HGC-27 cells. **(A)** CCK-8 assays showed that ISG20L2 knockdown significantly reduced cell viability and proliferation in AGS and HGC-27 cells compared with the control group. **(B)** Colony formation assays further demonstrated that ISG20L2 depletion markedly impaired the colony-forming ability of both cell lines. Statistical significance was indicated as ns, not significant (P ≥ 0.05); **P < 0.01; and ****P < 0.0001.

**Figure 12:**
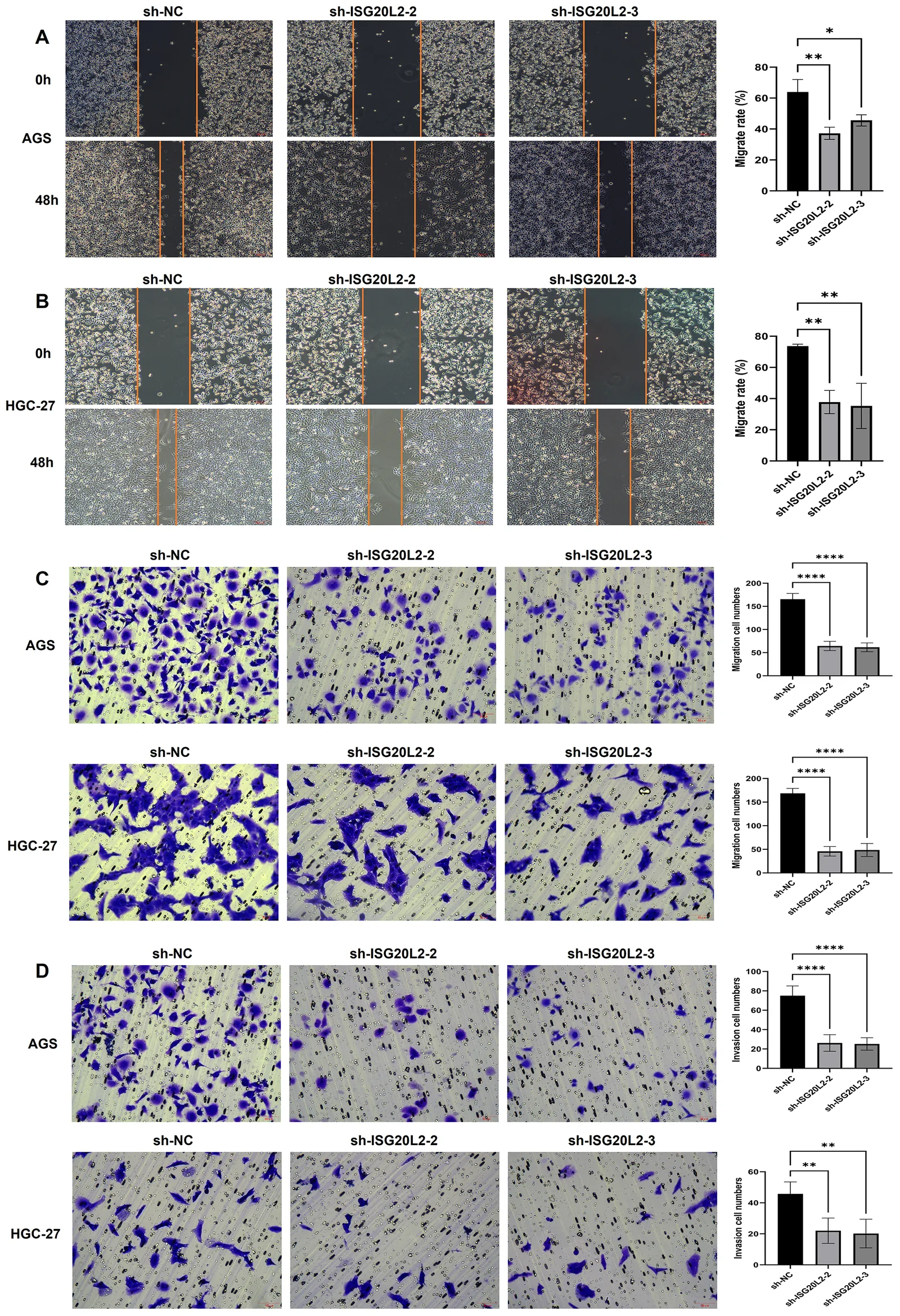
ISG20L2 knockdown inhibits the migratory and invasive abilities of AGS and HGC-27 cells. **(A, B)** Wound-healing assays showed that ISG20L2 knockdown significantly reduced the migratory capacity of AGS and HGC-27 cells compared with the control group. **(C, D)** Transwell assays further demonstrated that depletion of ISG20L2 markedly impaired the migration and invasion abilities of both cell lines. Statistical significance was indicated as ns, not significant (P ≥ 0.05); *P < 0.05; **P < 0.01; and ****P < 0.0001.

## 4 Discussion

This study identifies ISG20L2 as a potential pro-oncogenic regulator in gastric cancer. By integrating multi-omics analysis, single-cell transcriptomic profiling, clinical validation and *in vitro* functional assays, we demonstrated that ISG20L2 is markedly upregulated in gastric cancer and is closely associated with MSI status, promoter methylation, cell-cycle activity and immune microenvironmental features. Importantly, ISG20L2 knockdown significantly impaired the proliferation, colony formation, migration and invasion of AGS and HGC-27 cells, indicating that its aberrant expression has functional consequences rather than representing a merely correlative molecular alteration. Given the established role of ISG20L2 in pre-rRNA processing, ribosome biogenesis and T-cell regulation (16, 17, 21, 22), our findings suggest associations among ISG20L2, ribosomal homeostasis, cell-cycle activity, immune features and gastric cancer progression. These results extend the biological relevance of ISG20L2 in solid tumors and support further investigation of its potential clinical relevance in gastric cancer.

Based on the observed association between ISG20L2 upregulation, enhanced cell-cycle activity and increased malignant phenotypes, we hypothesize that ISG20L2 may promote gastric cancer progression by sustaining ribosome biogenesis and cell-cycle progression. This hypothesis is biologically plausible. ISG20L2 has been identified as a nucleolus-localized 3′–5′ exoribonuclease involved in the maturation of 5.8S rRNA, 18S pre-rRNA and 28S rRNA, thereby contributing to ribosome biogenesis (17, 22, 23). Ribosome biogenesis is a fundamental process required for cell growth and proliferation (24–26), and cancer cells frequently rely on increased rRNA processing and ribosomal assembly to meet the biosynthetic demands imposed by rapid expansion (27–29). In the present study, ISG20L2 knockdown markedly suppressed proliferation, colony formation, migration and invasion in AGS and HGC-27 cells, suggesting that ISG20L2 may contribute to these malignant phenotypes. This finding is consistent with the report by Ma et al. (17), which demonstrated that ISG20L2 promotes colon cancer cell proliferation and may do so through its role in ribosome biogenesis, and is also supported by Fang et al., who showed that ISG20L2 enhances proliferation and invasion in lung adenocarcinoma (18). Thus, ISG20L2 upregulation may confer functional advantages through pre-rRNA processing, ribosome production and cell-cycle progression, although this proposed mechanism requires direct validation. Beyond its potential role in tumor-intrinsic growth regulation, ISG20L2 may also influence the immune microenvironment through its function in ribosome biogenesis and post-transcriptional regulation. Given that enhanced ribosome production increases the translational capacity of rapidly proliferating tumor cells, ISG20L2 overexpression may facilitate the synthesis of immunomodulatory proteins, including secreted cytokines and immune checkpoint-related molecules. One possible mechanism is that ISG20L2-driven ribosome biogenesis promotes the translation of immunosuppressive mediators such as TGF-β and IL-10, thereby impairing CD8^+^ T-cell activation and contributing to an exhausted immune phenotype. Alternatively, ISG20L2 may regulate PD-L1 expression through RNA processing or translational control mechanisms, leading to enhanced immune evasion. Although these hypotheses remain to be experimentally validated, they provide a plausible mechanistic link between ISG20L2-mediated ribosomal regulation and the immune alterations observed in gastric cancer.

Nevertheless, the biological effect of ISG20L2 appears to be tumor-type and context dependent. Yang et al. reported that ISG20L2 knockdown did not substantially affect basal growth in multiple myeloma cell models, but instead increased sensitivity to proteasome inhibitors such as bortezomib and carfilzomib (30) This differs from our observation that ISG20L2 depletion directly impaired malignant phenotypes in gastric cancer cells. Such discrepancy may be explained by differences in tumor origin, cellular metabolic dependency and experimental context. Multiple myeloma is a hematological malignancy in which ISG20L2 was mainly investigated in relation to drug response and proteasome inhibitor resistance, whereas gastric cancer cells may rely more heavily on ribosome biogenesis, cell-cycle activation and invasion-associated programs to sustain their basal malignant behavior. In addition, our study revealed associations between ISG20L2 and immune infiltration, PD-L1 expression, and exhausted CD8+ T cells. These findings are consistent with recent evidence linking T-cell infiltration, PD-1/PD-L1 immune-checkpoint signaling, and CD8+ T-cell exhaustion to the tumor immune microenvironment, and are further supported by Rodríguez-Galán et al. (21), who identified ISG20L2 as an RNA nuclease involved in T-cell activation and the regulation of immunoregulatory molecules, including CTLA-4, TIM-3, PD-L1, and PD-1 (21, 31–33). Therefore, ISG20L2 may exert a dual role in gastric cancer: promoting tumor-intrinsic malignant progression through ribosome biogenesis and cell-cycle regulation, while also participating in immune microenvironment remodeling by influencing immune-regulatory programs and T-cell functional states. This mechanistic framework provides a plausible explanation for the observed associations of ISG20L2 with MSI status, promoter methylation and immunotherapy-related features. From a translational perspective, the expanding diversity of gastric cancer biomarkers supports a multimarker rather than a single-marker strategy. Non-coding RNA signatures can potentially provide minimally invasive information from blood or other biofluids, whereas protein-related markers can reflect tumor phenotype, invasion, or immune status, and emerging biosensing platforms may improve analytical sensitivity for early or dynamic detection (8–15). Within this framework, ISG20L2 may reflect ribosome biogenesis, proliferative, and immune-associated tumor states rather than serve as a stand-alone prognostic biomarker. Survival analyses showed no consistent association between ISG20L2 expression and DFI, DSS, OS, or PFI. Although an optimized cutoff yielded a nominally significant difference in OS, this finding was not reproduced by quartile-based stratification or across other survival endpoints. This does not contradict the biological role of ISG20L2, as a molecular regulator may influence tumor proliferation, invasion, or immune regulation without independently predicting patient survival, which is affected by multiple clinical and molecular factors. Moreover, analyses across molecular subtypes, pathological stages, and lymph node metastatic states were limited by incomplete clinicopathological and follow-up data in public datasets. Therefore, the prognostic and clinical stratification value of ISG20L2 remains uncertain and requires validation in larger, well-annotated gastric cancer cohorts and independent clinical samples. Several limitations should be acknowledged. First, part of the evidence was derived from public datasets and bioinformatic prediction. Although these findings were supported by clinical specimens and *in vitro* validation, the limited clinical sample size, including only nine paired gastric cancer tissues and adjacent non-tumor tissues, as well as potential heterogeneity in sample sources, sequencing platforms, and analytical pipelines, may still influence the interpretation of the results. Therefore, larger multicenter clinical cohorts are required to further validate the diagnostic and prognostic value of ISG20L2. Second, although public datasets allowed us to explore the molecular and immune associations of ISG20L2, the present study did not comprehensively evaluate its expression patterns across distinct molecular subtypes, pathological stages and lymph node metastatic states of gastric cancer. Therefore, whether ISG20L2 can serve as a robust stratification marker for specific clinical subgroups remains unclear. Future studies involving larger cohorts with detailed clinicopathological annotation are required to clarify these associations. Third, functional validation was performed mainly in two gastric cancer cell lines (AGS and HGC-27), which may not fully represent the molecular heterogeneity of gastric cancer. Although consistent inhibitory effects on proliferation, colony formation, migration and invasion were observed, the absence of an *in vivo* tumor model prevents us from determining whether ISG20L2 depletion can suppress gastric cancer growth in an organismal context. Therefore, the present findings support a pro-oncogenic role of ISG20L2 *in vitro*, whereas its *in vivo* contribution to tumor progression and its therapeutic relevance remain to be established. Future studies should directly investigate the mechanistic link between ISG20L2 and immune modulation. First, co-culture experiments using ISG20L2-overexpressing or silenced gastric cancer cells together with CD8^+^ T cells could determine whether ISG20L2 alters T-cell cytotoxicity, activation status and exhaustion-associated markers, including PD-1, LAG3 and TIGIT. Second, transcriptomic sequencing (RNA-seq) and ribosome profiling after ISG20L2 depletion would help identify downstream genes and translational targets responsible for its oncogenic and immunoregulatory effects. Third, *in vivo* validation using xenograft or immunocompetent syngeneic gastric cancer models will be required to determine whether ISG20L2 regulates tumor growth, immune-cell infiltration and response to immunotherapy within a physiological context. In addition, larger multicenter clinical cohorts with comprehensive molecular annotation and longitudinal follow-up are needed to validate the clinical significance of ISG20L2. Despite these limitations, the consistency among multi-omics analyses, protein-level validation and functional assays supports the conclusion that ISG20L2 is associated with gastric cancer progression and shows pro-oncogenic activity *in vitro*.

## 5 Conclusions

In conclusion, this study systematically demonstrates the aberrant expression, potential pro-oncogenic role and immune relevance of ISG20L2 in gastric cancer. Our findings indicate that ISG20L2 is associated with molecular heterogeneity, cell-cycle activity and immune microenvironmental features, while its knockdown significantly suppresses proliferation, colony formation, migration and invasion in gastric cancer cells. Together with previous evidence linking ISG20L2 to rRNA maturation, ribosome biogenesis and T-cell regulation, our study connects ISG20L2 with malignant progression and immune modulation in gastric cancer. These findings expand the biological understanding of ISG20L2 in solid tumors and support further investigation of ISG20L2 as a potential molecular indicator associated with gastric cancer biology, while its therapeutic relevance requires mechanistic and *in vivo* validation. Future prospective studies should further determine whether longitudinal or multiplex assessment of ISG20L2 together with established and emerging molecular biomarkers can improve evaluation of disease progression, treatment response, and clinical prognosis.

## Data Availability

The raw data supporting the conclusions of this article will be made available by the authors, without undue reservation.
