# Supplementary material for: Multi-omics and experimental validation reveal ISG20L2 as a potential pro-oncogenic regulator in gastric cancer
Source: Front Oncol. 2026 Sep 16;16:1906513. doi: 10.3389/fonc.2026.1906513 (PMC13623601; doi:10.3389/fonc.2026.1906513)
Supplement: Supplementary file 1 [file SupplementaryFile1.docx]

**Supplementary Fig. 1B analysis code:** CGA-only pan-cancer analysis demonstrated significant differences in ISG20L2 expression between tumour and adjacent non-tumour tissues.

**Analysis code:** source("/path/to/Sparkle.R")

library(tidyverse)

library(stringr)

library(dplyr)

library(ggplot2)

library(RColorBrewer)

library(data.table)

library(tibble)

data = getPanGeneZscore(GeneName)

data = data[which(data[,"CancerType"] %in% tumors),]

data = data[which((data[,GeneName] > -3) & (data[,GeneName] < 3)),]

data_clinical = table(data$CancerType,data$Type) %>% as.data.frame()

data_clinical_delete = data_clinical[which(data_clinical$Freq < 3),]

data = data[which(data[,"CancerType"] %!in% unique(data_clinical_delete$Var1)),]

write.csv(data,"_mRNA_PanCancer_zscore_TCGA_wilcox.csv")

pvalues = sapply(unique(data$CancerType), function(x) {

res = wilcox.test(as.numeric(expr) ~ Type, data = subset(data, CancerType == x))

res$p.value

})

pv = data.frame(gene = unique(data$CancerType), pvalue = pvalues)

pv$sigcode = cut(pv$pvalue, c(0,0.0001, 0.001, 0.01, 0.05, 1),

labels=c('****','***', '**', '*', '')

)

**Supplementary Fig. 1C analysis code:** Integrated TCGA-GTEx analysis, after tissue-specific correction of TCGA-versus-GTEx source effects, showed differential ISG20L2 expression between tumour and normal tissues.

**Analysis code:** source("/path/to/Sparkle.R")

data = readRDS(paste0("/path/to/TCGA_GTEx_",GeneName,".RDS"))

data = data[which(data[,"CancerType"] %in% tumors),]

data = data[which((data[,"Zscore"] > -3) & (data[,"Zscore"] < 3)),]

data_clinical = table(data$CancerType,data$Type) %>% as.data.frame()

data_clinical_delete = data_clinical[which(data_clinical$Freq < 3),]

data = data[which(data[,"CancerType"] %!in% unique(data_clinical_delete$Var1)),]

write.csv(data,"_mRNA_PanCancer_zscore_TCGA_GTEx_wilcox.csv")

#两组，wilcox.test或t.test；多组，kruskal.test或aov(one-way ANOVA test)

pvalues = sapply(unique(data$CancerType), function(x) {

res = wilcox.test(as.numeric(Zscore) ~ Type, data = subset(data, CancerType == x))

res$p.value

})

pv = data.frame(gene = unique(data$CancerType), pvalue = pvalues)

pv$sigcode = cut(pv$pvalue, c(0,0.0001, 0.001, 0.01, 0.05, 1),

labels=c('****','***', '**', '*', '')

)

**Supplementary Figure S1. Kaplan–Meier survival analyses of ISG20L2 expression in gastric cancer.**

(A–D) Kaplan–Meier analyses of disease-free interval (DFI), disease-specific survival (DSS), overall survival (OS), and progression-free interval (PFI), respectively, after patients were stratified into four groups (Q1–Q4) according to quartiles of ISG20L2 expression. No significant differences were observed among the four expression groups for DFI (log-rank P = 0.681), DSS (P = 0.528), OS (P = 0.329), or PFI (P = 0.369). Pairwise log-rank P values between quartile groups are shown in the corresponding matrices, and the numbers at risk are displayed below each Kaplan–Meier plot.

(E–H) Kaplan–Meier analyses of DFI, DSS, OS, and PFI, respectively, using optimized cutoffs to stratify patients into high- and low-ISG20L2 expression groups. No statistically significant differences were observed for DFI (P = 0.063), DSS (P = 0.067), or PFI (P = 0.102), whereas a nominally significant difference was observed for OS (P = 0.044). However, the OS association was not reproduced in the quartile-based analysis. Overall, these results indicate that the association between ISG20L2 expression and survival outcomes is not consistent across different stratification approaches and requires further validation in independent clinical cohorts. Tick marks indicate censored observations.
