# Supplementary material for: Multi-omics and experimental validation reveal ISG20L2 as a potential pro-oncogenic regulator in gastric cancer
Source: Front Oncol. 2026 Sep 16;16:1906513. doi: 10.3389/fonc.2026.1906513 (PMC13623601; doi:10.3389/fonc.2026.1906513)
Supplement: Supplementary file 2 [file Table1.docx]

**Figure 8** Raw western blot images demonstrate increased ISG20L2 protein expression in gastric cancer. (A) Western blot analysis showed that ISG20L2 protein expression was significantly higher in gastric cancer cell lines than in the normal gastric epithelial cell line GES-1.

**Figure 8** Raw western blot images demonstrate increased ISG20L2 protein expression in gastric cancer. (B) Western blot analysis of 9 paired STAD and adjacent non-tumour tissues further confirmed that ISG20L2 protein expression was elevated in tumour tissues.
